# Impact of mealtime social experiences on student consumption of meals at school: a qualitative analysis of caregiver perspectives

**DOI:** 10.1017/S1368980024002349

**Published:** 2025-02-04

**Authors:** Leah Elizabeth Chapman, Wendi Gosliner, Deborah A Olarte, Monica Daniela Zuercher, Lorrene D Ritchie, Dania Orta-Aleman, Marlene B Schwartz, Michele Polacsek, Christina E Hecht, Kenneth Hecht, Anisha I Patel, Punam Ohri-Vachaspati, Margaret Read, Juliana FW Cohen

**Affiliations:** 1 Department of Health Sciences and Nutrition, Center for Health Inclusion, Research, and Practice, Merrimack College, 315 Turnpike St, North Andover, MA 01845, United States; 2 Department of Nutrition, Harvard T.H. Chan School of Public Health, 655 Huntington Ave, Boston, MA 02115, United States; 3 Nutrition Policy Institute, Division of Agriculture and Natural Resources, University of California, 1111 Franklin St, Oakland, CA 94507, United States; 4 Department of Nutrition and Food Studies, New York University Steinhardt School of Culture, Education, and Human Development, 411 Lafayette Street, 5th Floor, New York, NY 10003, United States; 5 Rudd Center for Food Policy and Health & Department of Human Development and Family Sciences, University of Connecticut, One Constitution Plaza, Suite 600, Hartford, CT 06103, United States; 6 Center for Excellence in Public Health, University of New England, 1075 Forest Avenue, Suite 123, Portland, ME 04103, United States; 7 Stanford Pediatrics, Stanford University, 3145 Porter Drive, F110, Palo Alto, CA 94304, United States; 8 College of Health Solutions, Arizona State University, 550 N 3rd St, Suite 501, Phoenix, AZ 85004, United States; 9 Partnership for a Healthier America, P.O. Box 1200, Prince Frederick, MD 20678, United States

**Keywords:** School meals, Socialising, Social anxiety, COVID-19, Qualitative research

## Abstract

**Objective:**

To understand caregivers’ perceptions about their children’s mealtime social experiences at school and how they believe these social experiences impact their children’s consumption of meals at school (both meals brought from home and school meals).

**Design:**

Qualitative data were originally collected as part of a larger mixed methods study using an embedded-QUAN dominant research design.

**Setting:**

Semi-structured interviews were conducted with United States (U.S.) caregivers over Zoom^TM^ in English and Spanish during the 2021–2022 school year. The interview guide contained 14 questions on caregivers’ perceptions about their children’s experiences with school meals.

**Participants:**

Caregivers of students in elementary, middle and high schools in rural, suburban and urban communities in California (*n* 46) and Maine (*n* 20) were interviewed. Most (60·6 %) were caregivers of children who were eligible for free or reduced-price meals.

**Results:**

Caregivers reported that an important benefit of eating meals at school is their child’s opportunity to socialise with their peers. Caregivers also stated that their child’s favourite aspect of school lunch is socialising with friends. However, some caregivers reported the cafeteria environment caused their children to feel anxious and not eat. Other caregivers reported that their children sometimes skipped lunch and chose to socialise with friends rather than wait in long lunch lines.

**Conclusions:**

Socialising during school meals is important to both caregivers and students. Policies such as increasing lunch period lengths and holding recess before lunch have been found to promote school meal consumption and could reinforce the positive social aspects of mealtime for students.

The United States Department of Agriculture’s National School Lunch Program and School Breakfast Program provide healthful meals to approximately 30 million children in the United States (U.S.) each school day at low or no cost^(1,2)^. Students from households earning less than 130 % of the federal poverty guideline are eligible to receive free school meals, and those from households earning between 130 and 185 % of the federal poverty guideline can receive meals at a reduced cost^(2)^. All other students pay full price for school meals, but as all school meals are federally subsidised, full-priced meals are low cost (i.e. a national average of $1·75 for elementary and middle schools and $1·80 for high schools for breakfast and $2·83, $3·00 and $3·05 for lunch for elementary, middle and high schools, respectively, in 2024)^(1,2)^. The Healthy, Hunger-Free Kids Act of 2010 authorised the U.S. Department of Agriculture to update school meal nutrition standards to reflect the current Dietary Guidelines for Americans, making U.S. school meals on average healthier than meals brought from home and the healthiest source of nutrition for many U.S. children each day^(3–8)^. However, prior to the COVID-19 pandemic, only 80 % of students eligible for free meals and 67 % of students eligible for reduced-price meals participated in school lunch^(9)^. Participation among those who paid full price because of their higher household income was even lower (37 %)^(9)^.

Numerous factors influence school meal consumption, such as student demographics, food preferences and perceived stigma regarding school meals^(10)^. Mealtime operations (such as lunch period lengths, the number of lunch lines, the timing of recess and other school district policies and practices) can also influence students’ school meal participation and consumption of meals at school^(10)^. Additionally, mealtime socialising or ‘meeting and spending time with people in a friendly way in order to enjoy oneself’^(11)^ and the cafeteria social environment (e.g. crowding, noise level) may influence students’ consumption of school meals through numerous pathways. For example, socialising during meals may foster social support, thereby decreasing stress and allowing children to comfortably enjoy meals^(12,13)^. Socialising may also encourage children to try new foods (e.g. if a school is serving a new food that a child has not tried, a child may want to try the new food if they see their friends eating it)^(14)^. However, socialising could also divert children’s attention away from eating (e.g. more time spent talking rather than eating), thereby leading to less consumption^(15–17)^, particularly if children do not have enough time to eat. Additionally, some children may feel anxious about socialising during meals or may feel anxious in a crowded environment, which may lead to stress, anxiety and a reduced desire to eat meals^(18)^. Figure 1 displays these various proposed causal pathways.

**Figure 1.**
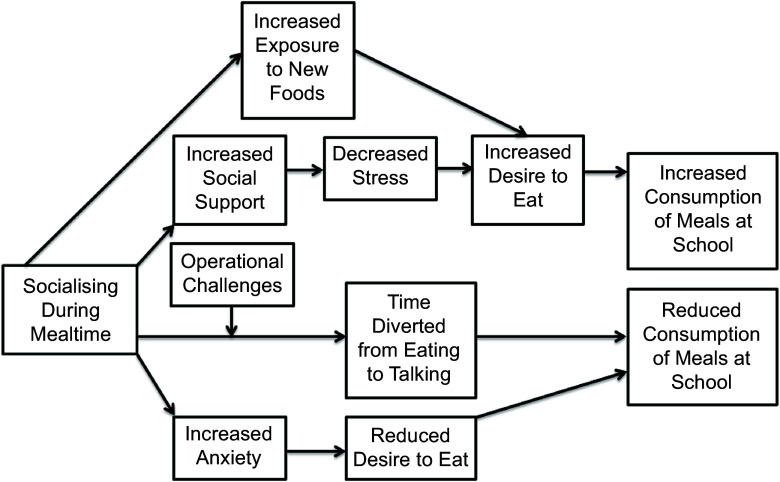
Conceptual model displaying the hypothesised relationship between socialising during mealtime and consumption of meals at school.

It has been hypothesised that school meals are important for building peer relationships, trust and social support^(19–21)^, but there is currently limited evidence documenting parents’ and caregivers’ (i.e. the person who plays the lead parental role, herein referred to as caregivers) perceptions of the importance of their children’s school meal social experiences, and whether these experiences impact consumption of meals at school. Understanding caregiver perceptions may be particularly important, as caregivers are often powerful change agents in school settings and serve as advocates for students. Understanding caregiver perceptions is also important because caregiver perceptions of school meals are positively associated with school meal participation rates^(22,23)^. Several studies have assessed parent perceptions of school meals generally, finding both negative or mixed views about school meal quality or healthfulness^(22–26)^. Prior research has also documented parental views on the perceived benefits of school meals including saving families money, time and stress^(23,24)^. However, to the authors’ knowledge, no studies have examined caregiver perceptions of school meal social experiences. Additionally, several studies have found that school mealtime social experiences (e.g. social support, peer influence) are associated with the consumption of meals at school^(27–30)^, but no studies have examined how or why this association may exist. Given these research gaps, this study aimed to understand caregivers’ perceptions about their children’s mealtime social experiences at school and how they think these might impact their children’s consumption of meals at school (both meals brought from home and school meals). The study used a qualitative approach that involved interviewing caregivers of elementary, middle and high-school children in the U.S. during the 2021–2022 school year.

## Methods

The present study is a secondary analysis of qualitative data that was collected as part of a larger mixed methods study examining the impact of the U.S. COVID-19 federal Universal Free School Meals (UFSM) policy on school meal participation rates and household food insecurity. The study also examined caregiver perceptions of school meals and related policies, including students’ experiences during school meals. California and Maine were the two states selected for this study because they were among the first U.S. states to adopt UFSM statewide once the federal policy ended after the 2021–2022 school year^(31,32)^. However, data for this study were collected when school meals were free of charge for all students due to the COVID-19 federal UFSM policy and prior to the implementation of California and Maine’s state-level UFSM policies. This mixed methods study used an embedded-QUAN dominant research design; the qualitative data were used to help contextualise and further explain findings from the quantitative results. As such, participants were first recruited for the quantitative portion of the study. All participants were recruited from the Galloway Research proprietary research panel. This panel contains more than one million U.S. residents who were recruited using a variety of methods, including online, television and radio advertising, corporate partnerships and word of mouth. More details on this research panel are published elsewhere^(23,24,33)^.

### Study population

To be eligible for the mixed methods study, caregivers were required to (1) live in the state of Maine or California; (2) be the caregiver of one or more children in grades kindergarten through twelve; (3) have a child who attended a public or charter school; and (4) have a child who attended either an elementary, middle or high school. In Maine, only schools that did not previously provide UFSM before the federal COVID-19 policy through programmes such as the Community Eligibility Provision were considered, but this was not the case in California. In California, policy requires schools that are eligible for Community Eligibility Provision to provide UFSM through Community Eligibility Provision^(34)^. Therefore, to obtain the necessary amount of lower-income households in the sample to be reflective of state demographics, the authors did not exclude Community Eligibility Provision schools from the California sample. Additionally, target sampling quotas for free and reduced-price meals eligibility and race/ethnicity were established to be representative of student population in each state^(35–38)^. More details on this study’s sampling quotas are published elsewhere^(24)^.

Participants eligible for the mixed methods study within the Galloway Research panel received an invitation with a survey link via email and text message inviting them to complete a survey about their opinions of school meals during the school year 2021–2022 (*n* 152 000). Out of these participants, 3202 accepted the invitation. However, 2012 participants did not meet the study’s eligibility criteria, or their free and reduced-price meals and/or race/ethnicity quotas were already met. The remaining 1190 participants (*n* 80 Maine participants and *n* 1110 California participants) completed the full survey. Due to California’s larger population and greater diversity by race/ethnicity, more survey responses were collected from caregivers in California than in Maine. Subsets of these participants (*n* 80 in Maine and *n* 220 in California) were randomly selected and invited to complete qualitative interviews to help contextualise findings from the surveys.

Trained researchers conducted semi-structured Zoom (Zoom Video Communications) interviews with caregivers in Maine and California in the late spring and early summer of 2022 (April, May and June). Out of the 80 caregivers in Maine and 220 caregivers in California invited to participate, 20 and 46 agreed to participate, respectively (*n* 66 caregivers total). More interviews were needed in California than Maine because of the greater diversity by race/ethnicity. The sample was evenly distributed by school type (with similar numbers of caregivers in elementary, middle and high school) and urbanicity (caregivers in rural, urban and suburban schools).

### Interviews

To develop the interview guide, the study researchers partnered with U.S. nutrition and school food policy advocacy organisations (including the School Nutrition Association (www.schoolnutrition.org), Center for Science in the Public Interest (www.cspinet.org), and the Urban School Food Alliance (www.urbanschoolfoodalliance.org), Full Plates Full Potential (www.fullplates.org) and Share our Strength (www.shareourstrength.org)), the California Department of Education (www.cde.ca.gov), and community school meal stakeholders. The research team pilot-tested the interview guide among socioeconomically and racially/ethnically diverse caregivers in both California and Maine and updated it based on the feedback. The final interview guide is provided in online Supplemental Figure 1.

The interview guide contained 14 questions on caregivers’ perceptions of school meals and their perceptions of their child’s experiences with school meals, as well as their opinions of the federal COVID-19 UFSM policy. While none of the questions focused specifically on social experiences during school meals, many caregivers’ responses to questions such as ‘What does your child like best about their experience with school lunch? What do they like least?’ or ‘What do you think are some of the benefits of schools providing lunch?’ included answers related to students’ social experiences during school breakfast and lunch. Thus, multiple themes around mealtime social experiences were prominent within the data. The research team therefore chose to examine these themes in the present study.

All interviews were recorded and conducted by five women research assistants trained in qualitative methods. Two data collectors were coauthors of the present study (one postdoctoral research fellow with a PhD in Nutrition and one research project manager with a master’s degree in public health), and the other three were bilingual professionals at Galloway Research. All data collectors had extensive experience working on various public health research projects throughout the United States. No data collectors had children who attended any of the schools in the present study. For this qualitative study, the data collectors’ backgrounds may have influenced the data collection and analysis. However, the data collectors and study authors are experienced in qualitative research methods, and numerous steps were taken to ensure credibility, confirmability and authenticity with the data (e.g. prolonged engagement with participants during the interviews, iterative questioning of the data, negative case analysis, peer-debriefing, keeping detailed notes of all coding decisions and analysis, and discussing these notes and decisions in peer-debriefing sessions with other experienced qualitative researchers)^(39)^. Maine interviews were conducted in English only; approximately one-quarter of the California interviews were conducted in Spanish by the three data collectors from Galloway Research who are fluent Spanish speakers. Interviews lasted between 30 and 40 min. No one else was present besides the participant and interviewer on the Zoom call during the interview. Two of the data collectors used Zoom’s transcription service to transcribe the interviews and then de-identified the transcripts and checked the transcriptions for accuracy. Each caregiver provided written consent and completed a brief survey before participating in the interview. The Merrimack College Institutional Review Board and the Institutional Review Board of the University of California, Davis approved this study.

### Data analysis

Using thematic analysis to code and analyse the qualitative data, the lead author first reviewed a set of 13 transcripts (five in Maine and eight in California) and developed codes and sub-codes. Then, using immersion-crystallisation methodology (an inductive, iterative process for identifying themes, categories and patterns in qualitative data^(40)^), the lead author and one research assistant formally coded the 13 interviews and refined the codebook to further reflect the transcripts’ content. A total of 28 codes and 21 sub-codes were created (nine of which were codes regarding the social experiences of mealtime). Five additional transcripts were subsequently coded and cross-checked using the finalised codes until inter-rater reliability reached at least 75 % (calculated using the percent agreement (the number of times the coders agreed on the same data item, divided by the total number of data items))^(41)^. The lead author and research assistant then coded the remaining 48 transcripts with the 49 finalised codes using Excel® version 14.0.0. Thematic saturation across the broader study was reached (i.e. no new information was identified) after 30 interviews; however, all remaining interviews were reviewed and coded with the 49 codes. (Had new information been identified, the lead author and research assistant would have revisited and updated the codebook with new codes.) Codes were then grouped to create themes. From the themes, the lead author (with assistance from senior members of the research team) generated domains that encapsulated and summarised the main outcomes from the study. The broader mixed methods study’s primary aims were to examine the impact of the U.S. COVID-19 federal UFSM policy on school meal participation rates and household food insecurity, and themes related to these topics were first examined and published earlier^(23,24)^. After publishing these findings, other findings (such as the findings in the current study on mealtime social experiences) were subsequently examined.

## Results

Table 1 presents the characteristics of the caregiver interviewees. Most caregivers interviewed in California and Maine were women, had children who were eligible for free and reduced-price meals and lived in households with three to five people. On average, caregivers had children in an even distribution of schools (i.e. elementary, middle school or high school), but there was a slightly larger sample of students identifying as boys in both California and Maine compared to the general population. All caregivers in Maine were white and non-Hispanic/Latino, which reflects Maine’s statewide demographics^(35,36)^. Reflective of the demographics of California, approximately half of the caregivers were of Hispanic/Latino origin^(37,38)^.

**Table 1.** Demographic characteristics of 66 caregivers of public or charter school students in grades kindergarten through twelve who participated in qualitative interviews on caregivers’ perceptions of school meals in Maine (*n* 20) and California (*n* 46) during the 2021–2022 school year

Sample characteristics	Maine	California
	*n*	*n*
Total sample	20	46
Caregiver’s gender		
Man	2	4
Woman	18	42
Household size^*^		
Two^†^	1	6
Three	7	14
Four	5	18
Five	4	5
Six	0	2
Seven	3	1
Free or reduced-price meal eligibility		
Free	10	19
Reduced	1	10
Near eligible	9	17
Child’s gender		
Boy	12	26
Girl	8	20
Child’s race		
White	20	26
Black or African American	0	6
Asian	0	6
American Indian or Alaska Native	0	2
Native Hawaiian or Other Pacific Islander	0	0
Other^‡^	0	6
Child’s ethnicity		
Hispanic/Latino	1	25
School type		
Elementary	9	16
Middle	5	14
High	6	16

* ‘Household size’ refers to the total number of individuals living in the household, which could be a combination of children and/or adults.

† ‘Two’ signifies one caregiver and one child.

‡ Includes participants who identified their race as ‘Other,’ ‘Mexican American’ or ‘Latino’.

Three domains were constructed through the analysis: (1) caregivers believe it is important for children to socialise during school mealtime, (2) socialising can be an incentive for consuming meals at school, and (3) socialising can be a barrier for consuming meals at school. For each domain, major themes and illustrative quotations are summarised in Table 2. All quotations are from different participants.

**Table 2.** Domains, themes and illustrative quotations from 66 caregivers of public or charter school students in grades kindergarten through twelve who participated in qualitative interviews on caregivers’ perceptions of school meals in Maine (*n* 20) and California (*n* 46) during the 2021–2022 school year

Domains and themes	Illustrative quotations
1. Caregivers believe it is important for children to socialise during school mealtime.	
1a. Caregivers believe the opportunity for their children to socialise with peers is an important benefit of school meals.	*‘I think just that it (school meals) helps the community…Plus, your kid getting a free lunch and being able to socialize, all that stuff.’* -Caregiver of a non-Hispanic/Latino white first-grade boy in California
1b. Caregivers and students disliked how COVID-19 social distancing protocols disrupted the social aspect of school meals for their children and were pleased when social distancing protocols were lifted.	*‘Um, the only the one big thing that we had an issue with during COVID, and as far as I know it’s all better now, but they couldn’t put all the kids in the cafeteria together, so (child’s name) was having to eat in the gym and that did not go over well with him. So, and they also they had to stay with their classmates. So he would be in the room and his friend would be like three tables away…’* -Caregiver of a Hispanic/Latino white eighth-grade boy in Maine *‘I would say now because they’re back, he’s back in person, it’s just the fact that he can sit with his classmates, with his friends, be back in that environment, the cafeteria and everyone together. I think that, for him, makes it a little better.’* -Caregiver of a Hispanic/Latino white eighth-grade boy in California
2. Socialising can be an incentive for consuming meals at school	
2a. Many students enjoy lunchtime, which creates a conducive setting for consuming their lunch.	*‘Her favorite thing about school lunch is being able to socialize with her friends…’* -Caregiver of a Hispanic/Latino white tenth-grade girl in California *‘But she really likes the fact that it’s (school lunch) like a time for all the students to kind of get together. She’s in social work for her social skills so, especially with her, they have like a lunch bunch, so she has a group of kids that she gets to select from her school around her and to sit and have lunch with them work on social skills so. That’s what she likes.’* -Caregiver of a non-Hispanic/Latino white sixth-grade girl in Maine
2b. Students like eating the same foods as their friends.	*‘So she likes that, and then she likes to be able to eat the same thing her friends are eating.’* -Caregiver of non-Hispanic/Latino Asian fourth-grade girl in California *‘So (child’s name) feels like he is able to make some food choices. And, also, eat what his friends are eating and be part of the ‘in crowd’ or whatever you want to say.’* -Caregiver of a Hispanic/Latino white tenth-grade boy in California *‘I think she enjoys that (when schools serve pizza for lunch), and it’s a day that all the other kids eat the same stuff. Her school offers a salad bar…and she eats a lot of vegetables…and her friends will comment on the fact that she eats ‘odd’ food because she’s eating a tomato. So I think she likes how inclusive the pizza days are. Like everybody is as excited about the food as she is, because you know, she’s over there with a salad with chickpeas on it and everyone’s like, “What are you doing?”’* -Caregiver of a non-Hispanic/Latino white third-grade girl in Maine *‘I would say her favorite part of it (school lunch) is the social aspect of it, because her friends are eating it too.’* -Caregiver of non-Hispanic/Latino Asian first-grade girl in California *‘…in Asian households, we have particular meals that I can give it to her, but then she would come and say, ‘Why do you give me this? My friends were eating a cheeseburger. You should give me something like that.’ So I know it’s (school lunch) a standard meal. Everyone is eating that, so they don’t have that. (inaudible 00:17:44) and I’m eating the other thing.’* -Caregiver of a non-Hispanic/Latino Asian first-grade girl in California
2c. Some students eat school breakfast so they can socialise with their friends.	*‘She eats breakfast there because she goes early to hang out with her friends.’* -Caregiver of a Hispanic/Latino white tenth-grade girl in California *‘So the days she eats breakfast, it’s because she gets ready early and she’s not in a mood to eat breakfast at home and mostly, ‘My friends are coming early.’ Not because we can’t prepare breakfast in the morning, but it’s mostly because, ‘I got up early, and I’m leaving for school.’ So our school is just two-minute walk from the house so she can get there early. And it’s mostly, you can talk to your friends during breakfast.’* -Caregiver of a non-Hispanic/Latino Asian first-grade girl in California
2d. Students’ friends encourage them to try new foods.	*‘The ability for her to try some new things with like positive people around her. Like to come home and be like ‘Oh my word, we had this for lunch and I wasn’t going to try it…’ but you know like her friends had it, so the growth that eating with other people brings is really nice.’* -Caregiver of a non-Hispanic/Latino white third-grade girl in Maine *‘I know my daughter says, ‘Oh, I don’t like that at home.’ But I hear that she’ll eat it at school. So, it’s probably, just a matter of my cooking. She doesn’t like on certain things, but when she’s with her friends, she feels maybe a little more pressured to eat the same things that they are.’* -Caregiver of a non-Hispanic/Latino white eighth-grade girl in California *‘…if they see different friends eating different things they are more apt to try it. And then they might come home and tell me ‘Hey I like this food’ or ‘I want that instead’ or something like that.’* -Caregiver of a non-Hispanic/Latino white sixth-grade girl in Maine
3. Socialising can be a barrier for consuming school meals	
3a. When schools have short lunch periods or long lunch lines, many students forego eating and choose to socialise instead.	*‘…I think the issue is they just want to play with their friends mostly. So they’ll grab it, sit down, pick a couple things off of it and leave. So then they get more time for their break, but he’s only eating the carrots.’* -Caregiver of a non-Hispanic/Latino white eighth-grade boy in California *‘And then like I said before, when they had all the fifth graders released at the same time waiting in line for lunch, he would complain like by the time he got his lunch, they did get time to sit down. But if others were leaving for recess, he’s not going to miss recess. So he would just ditch the lunch and then go to recess.’* -Caregiver of a non-Hispanic/Latino Asian fifth boy in California *‘My older daughter used to eat (school lunch) every day because she is a social butterfly… but the schedule at school makes it hard for her to get enough time to get through the lunch line from where her class is to the cafeteria, and then have time to eat. Some days she goes without eating school lunch because she doesn’t have time to actually get her lunch and eat it.’* -Caregiver of a non-Hispanic/Latino white eighth-grade girl in Maine *‘I think the little kids sometimes eat better, or sometimes they are playing and don’t want to eat. I think they could use a little more time.’* -Caregiver of a Hispanic/Latino ‘other race’ third-grade boy in California *‘Because she’s like, ‘It’s super crowded, mom.’ And it’s good because more kids are getting to eat and it’s social, for high schoolers anyway. But by the time they get through, it’s tough because they maybe have five minutes to eat after they’ve waited in line. And I think that’s the only daunting thing is kids sometimes don’t want to wait in line, they want to socialize with their friends and have a true break.’* -Caregiver of a non-Hispanic/Latino white tenth-grade girl in California *‘I think she would say she likes to hang out with her friends more than to eat. And so that’s why she probably doesn’t get it.’* -Caregiver of a non-Hispanic/Latino white seventh-grade girl in California
3b. Some students feel anxious because of the cafeteria social environment and therefore may not participate in or eat school lunch or eat a lunch packed from home.	*‘She doesn’t like making eye contact with the lunch lady…And then it’s, once you get your tray trying to figure out which social table you’re supposed to sit at, hoping you don’t take somebody else’s seat. So in reality you’re taking up four to five minutes of your twenty-minute lunch break, just trying to get over the anxiety of it. And then of course you’re, maybe you do like the food, but your anxiety is so bad you’re not going to eat.’* -Caregiver of a non-Hispanic/Latino white ninth-grade girl in Maine *‘Here, I have fruits and vegetables, things that she likes to eat, in small containers for her to bring with her, but she doesn’t want to take them. You see? Adolescents are at an age that sometimes they are embarrassed for their friends to see them at school with food from home. They don’t like that. But sometimes she doesn’t eat anything until she gets home.’* -Caregiver of a Hispanic/Latino white eighth-grade girl in California *‘Sometimes, he does tell me that he gets a little embarrassed, so he won’t eat. So on those days he goes to a teacher’s room and goes with his friends and they eat snacks, sometimes offered by the teacher because she’s concerned like, ‘Why aren’t you guys eating?’ They’re like, ‘Oh. We don’t feel like it.’ It’s not just my son, but some of his friends…And I’m not sure if other social things were happening also where they didn’t want to go to lunch because he’s not very open about those things. I tried but he won’t say.’* -Caregiver of a Hispanic/Latino ‘other race’ ninth-grade boy in California
3c. Caregivers suggested strategies to improve the social aspects of school meals and therefore consumption of school meals.	*‘Letting her go down like 5 min early when there isn’t a line…They used to have her leave a classroom early so she didn’t have to deal with all the people in the hall because it’s too much with anxiety.’* -Caregiver of a non-Hispanic/Latino white eighth-grade girl in Maine *‘So also maybe offering online for us to know where our kids are eating. Are they going to their teachers? What is a friendly place where they can go, not just a specific classroom, but like, ‘Hey. Students can eat foods in this room or at the gym or whatever.’ Sites where they can go.’* -Caregiver of a Hispanic/Latino ‘other race’ ninth-grade boy in California *‘When I was going to school, they literally scheduled sit down and eat time. You could not go and play with your friends until you were done eating your food. And that’s one of the things I think is missing right now is they’re giving kids an option to sit and eat or to not eat.’* -Caregiver of a non-Hispanic/Latino Asian kindergarten boy in California

### Domain 1: Caregivers believe it is important for children to socialise during school mealtime

#### Theme 1a: Caregivers believe the opportunity for their children to socialise with peers is an important benefit of school meals

Many caregivers stated that socialising was a benefit of schools providing meals to students (Table 2, Theme 1a). For example, one caregiver explained, ‘I think it’s good that they have it (school meals) because it’s a time for the kids to…sit and be able to eat and have some social time’ (caregiver of a non-Hispanic/Latino white second-grade girl in Maine). Additionally, caregivers appreciated that school meals brought students together and provided them with a shared meal experience. A caregiver stated, ‘And socialising, I think it’s important that they have access to food, and they can just sit down together and eat the same things and whatnot, and it gives them something common’ (caregiver of a non-Hispanic/Latino white eighth-grade girl in California).

#### Theme 1b: Caregivers and students disliked how COVID-19 social distancing protocols disrupted the social aspect of school meals for their children and were pleased when social distancing protocols were lifted

Many caregivers disliked the way that COVID-19 social distancing protocols disrupted socialising during mealtime at school, and they perceived that their children also disliked this (Table 2, Theme 1b). For example, a caregiver explained, ‘I think the community that a group meal brings…is really beneficial to just the mental state of everyone and the kids being able to…I know during COVID, only eating in your classroom with just your classroom was such a bummer. It was so celebrated…the community aspect of the school is eating lunch together…’ (caregiver of a non-Hispanic/Latino white third-grade girl in Maine). Additionally, some caregivers stated that the social distancing protocols made their children feel isolated and depressed. One caregiver stated, ‘In fact, during the pandemic, they had them (the students) more closed off, they restricted their social circle, and in my daughter’s case, she fell into a great depression’ (caregiver of a Hispanic/Latino white eighth-grade girl in California). At the time of this study’s data collection, some schools in California and Maine had eased or eliminated social distancing protocols, which pleased many caregivers and students. Caregivers stated that it was hard for their children to adhere to social distancing protocols, such as eating with the same ‘lunch buddy’ every day. One caregiver explained, ‘So the pandemic time…it was hard for her. It was hard always having to have lunch with the same buddy every single day. So one thing she has been happy with is being able to sit with new friends or to have different people at her table…’ (caregiver of a non-Hispanic/Latino white tenth-grade girl in Maine). Many caregivers were happy that students had returned to pre-pandemic congregate eating settings.

### Domain 2: Socialising can be an incentive for consuming meals at school

#### Theme 2a: Many students enjoy lunchtime, which creates a conducive setting for consuming their lunch

Many caregivers stated that their child’s favourite thing about lunchtime at school was socialising with their friends (Table 2, Theme 2a). A caregiver stated, ‘Hanging out with friends is probably the only reason why he’ll get interested in it (school lunch), yeah’ (caregiver of a white non-Hispanic/Latino sixth-grade boy in Maine). Several caregivers reported that their children could sit anywhere in the lunchroom (i.e. they did not have to sit with their classmates), enabling their children to socialise with other friends who were not in their class. One caregiver explained, ‘Well, of course what he likes the best is that he gets to hang out with his friends, or have a chance to sit and talk to kids from other classes that he don’t get a chance to see’ (caregiver of a non-Hispanic/Latino, black or African American second-grade boy in California). Overall, many caregivers reported that socialising contributed to positive perceptions of lunchtime for students, which created a conducive setting for consumption for both students who consumed school meals and for students who brought their lunch from home.

#### Theme 2b: Students like eating the same foods as their friends

Many caregivers reported that their children enjoyed eating school lunch because they ‘liked eating the same foods as their friends’ (Table 2, Theme 2b). One caregiver explained, ‘I do give her some snacks to take with her, but she likes to eat at school mainly because her friends like to eat it too, so they like to eat it together’ (caregiver of a non-Hispanic/Latino Asian first-grade girl in California). Additionally, some caregivers stated that eating the same meals as your friends made you part of the ‘in crowd.’ One caregiver of a non-Hispanic/Latino Asian first-grade girl in California also explained that eating school lunch reduced stigma for her daughter because she could eat the same foods as her friends, rather than bring food from home that was culturally different.

#### Theme 2c: Students eat school breakfast so they can socialise with their friends

Many caregivers reported that their child chose to eat school breakfast so they could socialise with their friends before school (Table 2, Theme 2c). One caregiver explained, ‘It’s (school breakfast) social. For her, it’s social. For other kids, it’s hunger. She has a little friend, in fact, I picked her up to go to the mall and she’s like, ‘Yeah, I go to school 07.30 every day. I’m part of the breakfast club’’ (caregiver of a non-Hispanic/Latino white tenth-grade girl in California). Several caregivers reported that their child would not eat school breakfast unless their friends specifically asked if they would accompany them. Other caregivers reported that their children would wake up earlier so they could arrive at school in enough time to eat breakfast with their friends.

#### Theme 2d: Students’ friends encourage them to try new foods

Many caregivers reported that their children were willing to try new foods during school lunch because of their friends’ encouragement (Table 2, Theme 2d). One caregiver stated, ‘Well, it’s (a packed lunch from home) his first option, but if they are serving something that he likes or a friend tells him to try it because it’s good, then he will try it’ (caregiver of a Hispanic/Latino ‘other race’ third-grade boy in California). Caregivers explained that their children would come home and excitedly recount what they tried at school and explained that their friends introduced them to the new food(s). This was primarily reported by caregivers of students in elementary school.

### Domain 3: Socialising can be a barrier for consuming school meals

#### Theme 3a: When schools have short lunch periods or long lunch lines, many students forego eating and choose to socialise instead

When mealtime operational challenges were reported, such as long lunch lines or short lunch periods, caregivers reported that their child would rather socialise with their friends than eat lunch (Table 2, Theme 3a). Some caregivers reported that to eat the school lunch, their child would have to wait in long lunch lines, and they would, therefore, miss out on social time with their friends. Instead, students would skip lunch in order to sit with their friends and socialise, even if this resulted in not eating at all. This behaviour was reported most frequently by caregivers of older girls. Some caregivers of younger students also reported that their child was so busy socialising that they did not have time to finish eating their lunch. Additionally, many caregivers reported that their child rushed through lunch (or did not finish their lunch) because they wanted to go outside or go to the gym to play and socialise with their friends during recess. Caregivers explained that many schools provide a combined set amount of time for both lunch and recess; therefore, the less time a child spends at lunch, the more time available for recess. This was especially an issue for students who waited in long lunch lines, even when students were eligible for free and reduced-price meals. By the time the students received their school lunch and sat down at the lunch table, many of their friends had finished eating and were leaving for recess. Caregivers explained that their child would, therefore, just eat one or two items and then throw the rest of their food away so they could join their friends for recess.

#### Theme 3b: Some students feel anxious because of the cafeteria social environment and therefore may not participate in or eat school lunch or eat a lunch packed from home

Not all students enjoyed socialising during lunch; some caregivers (especially caregivers of students in middle and high school) reported that their children felt anxious during lunchtime (Table 2, Theme 3b). Reasons included a crowded cafeteria environment, feeling insecure about where to sit, feeling shy about eating in front of other people and general anxiety about standing in the lunch line. A caregiver explained, ‘My younger daughter, her anxiety got worse during the pandemic. She doesn’t like to go up to the lunch line. So she’ll make every excuse in the book not to go to lunch and not to eat because of the crowds’ (caregiver of a non-Hispanic/Latino white eighth-grade girl in Maine). Some caregivers explained that their children were afraid of standing alone in the lunch line, but they would stand in the lunch line if they had a friend go with them. One caregiver reported that her child and his friends skip lunch and go sit in their classroom with their teacher; she thought this could be due to social anxiety. Overall, these reported anxieties led to students skipping lunch or eating less at lunch.

#### Theme 3c: Caregivers suggested strategies to improve the social aspects of school meals and therefore consumption of school meals

Caregivers suggested multiple strategies for overcoming the barriers that socialising poses to school meal consumption for their children (Table 2, Theme 3c). For example, in schools with combined lunch and recess, caregivers suggested that schools should allocate a separate amount of time for lunch and recess. This way, students will not rush through or skip lunch to get to recess. Additionally, some caregivers stated that their children should be able to eat in other places besides the cafeteria, for example, outdoors or in classrooms with smaller groups of students. One caregiver also suggested that students with anxiety should be allowed to leave for lunch five minutes early so they can obtain their lunch in a calmer and quieter environment and before the crowds arrive.

## Discussion

This study examined how caregivers perceive mealtime social experience using a qualitative approach that involved interviewing caregivers of students in California and Maine. The study’s primary finding was that caregivers perceive that peer socialising is an important benefit of school meals for their children. This is similar to findings from a 2022 qualitative study in Norway by Heim et al., which found that teachers and school administrators perceived that school meals provided important opportunities to create social relationships among students^(20)^. Similarly, results from a 2021 national series of discussion boards and surveys in the U.S. found that 77 % of 1018 low and middle-income caregivers thought school meals allowed their child to ‘build community and friendship with other students’^(25)^. Caregivers may perceive that peer socialising is an important benefit of school meals for numerous reasons. Mealtime peer socialising could improve their child’s social competency, allow their child to form deeper bonds with their classmates (which could facilitate cooperation and better learning in the classroom), or refresh children by providing more energy and better prepare them to learn during their afternoon classes^(12,42)^. Future research should investigate this further.

The present study also found that COVID-19 social distancing protocols disrupted the positive social aspects of lunch and caused some students to feel isolated and depressed. Other studies have found associations between social isolation during the COVID-19 pandemic more generally and depression among children^(43,44)^. Results from a U.S. COVID-19 longitudinal study of 133 children ages 4–11 found that child depression symptoms were highest following initial stay-at-home orders in North Carolina (April 2020), but then linearly decreased over a 15-month period^(43)^. Additionally, a meta-analysis of 53 longitudinal cohort studies found an increase in depression and anxiety symptoms during the COVID-19 pandemic among children and adolescents^(44)^. Overall, results from this study and previous studies suggest that if schools implement social distancing protocols in the future, they should carefully design these protocols to allow for as much social interaction as safely as possible (e.g. students eat together, but outside). This may help minimise feelings and symptoms of depression among students, while also encouraging consumption of meals at school.

Additionally, this study found that socialising can facilitate consuming meals at school. Caregivers reported that their children enjoyed socialising during school lunch and breakfast and that this positive view of school mealtime created a conducive setting for consuming their meals (both meals brought from home and school meals). This is similar to results from a U.S. repeated cross-sectional study among 20 third and fourth-grade classrooms across 6 schools by Blondin et al., which found that listening, working and/or socialising (compared with only eating) was associated with a 10 % reduction in milk waste among children who selected milk^(30)^. Additionally, in the present study, caregivers reported their students enjoy eating the same foods as their friends and will sometimes try new foods at school because of their friends’ encouragement (especially younger students). Mumm et al. similarly found that increased social support provided by ‘other kids at school’ increased support for school breakfast consumption^(26)^. Additionally, similar to the present study, a 2012 study by Bruening et al. found positive associations between adolescents and their friend groups and best friends for breakfast eating^(24)^. Also similar to the present study, a qualitative study of 47 adolescents eligible for the Supplemental Food and Nutrition Assistance Program ages 9–13 in Virginia, Hawaii and Montana found that students reported finishing their lunch when influenced to do so by their peers^(17)^. Overall, findings from the present study and the aforementioned studies suggest that socialising provides a conducive setting for consumption of meals at school; when children relax, eat and socialise without feeling rushed, they may eat more of their food, enjoy their food more and may even try new and/or healthier foods^(12)^. Additionally, role modelling or peer norms may be a mechanism for the relationship between socialising and consumption of meals at school, but future research should examine this further.

This study also found that students may prioritise socialising over eating and therefore, under specific circumstances (e.g. if time for lunch and recess are combined, time for lunch is inadequate or lunch lines are long), socialising may act as a barrier to consumption. These results are similar to results from a qualitative focus group study conducted among 64 adolescents in Los Angeles, which found that long cafeteria lines and time constraints were perceived barriers to eating school lunches^(45)^. Thus, schools should provide lunch periods that are long enough for students to walk to the cafeteria, wait in line and obtain their lunch, enjoy their meals and socialise with their friends. Previous studies demonstrate that having at least 20 min to eat lunch is associated with reduced plate waste and improved diet quality among students, and a lunch period of at least 30 min is likely to allow students to have this recommended 20 min of seated time^(46–48)^. Additionally, in the present study, caregivers of younger students reported that students prefer to play and socialise during recess, which causes them to rush through or reduce their consumption of lunch. Therefore, to prevent recess from acting as a barrier to school meal consumption, elementary schools could mandate time for lunch separate from the amount of time for recess to prevent students from rushing through lunch to arrive at recess faster. Further, elementary schools could schedule recess before lunch^(49,50)^.

Some caregivers, especially caregivers of older students, reported that the cafeteria social environment made students feel anxious and prevented them from consuming meals at school. This is similar to the previously described 2022 qualitative study of school teachers and administrators in Norway by Heim et al., which also found that some students feel anxious in the cafeteria environment (e.g. they feel anxious about finding a seat and deciding who to sit next to)^(20)^. For students who experience mealtime anxiety and dislike the crowds and noisiness of the cafeteria, schools could allow students to bring their meals outdoors (weather permitting), into the school library, guidance counsellor’s office or other settings such as empty classrooms for a quieter and less stressful experience.

This study had several strengths. First, this study relied on a large sample of qualitative interview data, which provided rich and useful descriptions of caregivers’ perspectives regarding their children’s social experiences during school meals. Additionally, the study population included caregivers of elementary, middle and high-school students; thus, potential differences in caregiver responses by grade were likely captured.

This study also had limitations. Although the study’s sample was not nationally representative, it did contain data from caregivers in two states that vary greatly in terms of geography, urbanicity and population demographics. Future studies should be conducted in a greater number of states to determine if the results from this study are generalisable to the greater U.S. population. Additionally, the data collection method relied on self-reporting from caregivers who may have limited or inaccurate knowledge of their children’s experiences during school meals. The research team did not ask students about socialising during school meals due to the study’s original design, target sample and research questions regarding caregiver perceptions of school meals and the COVID-19 federal UFSM policy. However, understanding caregiver perspectives on various aspects of school meals is valuable and important because caregivers often represent their children in organisations such as Parent Teacher Associations and events such as school board meetings (in which policies impacting students are often made). Additionally, understanding caregiver perspectives is important because caregiver perceptions of school meals are associated with school meal participation rates^(21)^. However, additional qualitative research conducted directly with students is warranted to ensure that caregivers are not perceiving their children’s mealtime social experiences at school inaccurately. Additionally, this study was a secondary analysis of qualitative data; the interview guide was not originally designed to answer this study’s research questions. While peer socialising during mealtime was not originally a primary focus of the study, this *was* a primary response from many caregivers when responding to questions from the interview guide. Future research studies should develop interview guides that specifically focus on this concept to determine if there are additional caregiver perceptions regarding the social aspects of school meals. An additional limitation is that while caregivers stated that it was important to them that their children have an opportunity to socialise during mealtime at school, they did not specifically state why. Future research should focus on school mealtime socialisation with detailed interview guides that can further probe about why caregivers find this important. Finally, data for this study were collected at the end of the COVID-19 pandemic, which could have affected the school meal operations and parental perceptions of mealtime at school. For example, peer socialising may have been deemed more important at lunchtime, since this may have represented one of the few opportunities for students to socialise during the pandemic. Future studies under ‘normal’ post-pandemic conditions are needed.

Overall, caregivers generally perceive that positive mealtime social experiences and social environments can promote the consumption of meals at school for their children. Strategies such as allowing enough time for both socialising and eating (at least 30 min) and holding recess before lunch may promote positive mealtime social experiences and increased school meal participation and consumption. An additional strategy that caregivers suggested (and that certain schools throughout the U.S. are implementing) included allowing students to eat in alternative locations (such as empty classrooms or libraries), although future research should determine whether these strategies are causally linked to improvements in mental health, social experience, school meal participation and meal consumption among students.

## Supporting information

Chapman et al. supplementary material Chapman et al. supplementary material

## Supplementary material

For supplementary material accompanying this paper visit https://doi.org/10.1017/S1368980024002349
