# Supplementary material for: Impact of mealtime social experiences on student consumption of meals at school: a qualitative analysis of caregiver perspectives
Source: Public Health Nutr. 2025 Feb 4;28(1):e55. doi: 10.1017/S1368980024002349 (PMC11983997; doi:10.1017/S1368980024002349)
Supplement: Chapman et al. supplementary material [file S1368980024002349sup001.pdf]

Supplemental Figure 1. Parent 14-question interview guide that was used to inform the present analysis of parents' perceptions mealtime social experiences in Maine (n=20) and California (n=46) during the 2021-2022 school year.

Background

1. What ages and grades are your kids in?
    a. How often does *[each/your child]* **currently** eat school breakfast, school lunch and/or any other meals or snacks served by school?
    b. **Prior to the COVID pandemic**, on average, how many days did *[each/your child]* eat school breakfast, school lunch and/or any other meals or snacks served by school?
        i. [*If different between years*] What do you think caused the change in eating school meals between now and before the COVID pandemic?
    c. *[If not already answered]* **Prior to COVID**, was your family eligible for free or reduced-price meals at school?

[If more than one child]

To make this interview as brief as possible, we will just talk about one of your children's experiences with school meals today. Please focus on your child with the most recent birthday. Do you mind sharing this child's first name, so we can then talk about them by name as we go? What grade is [*child's name*] in?

[If only one child]

Do you mind sharing your child's first name, so we can then talk about them by name as we go?

School Lunch

First, I'd like to talk about [*child's name*] experience with **just** the school lunch (we will talk about school breakfast later).

2. Does *[child's name]* talk about school lunch with you?
    [IF YES]
    a. What does *[child's name]* think of the meals?
        i. Probe about quality, quantity, and overall appeal
    b. *(if they eat school lunch)* What do they like best about their experience with school lunch? What do they like least?
        i. Probe about stigma, convenience, cafeteria environment, timing of the meals
    c. (*If they eat school lunch*) Can you talk about which school lunch meals your child likes the most and why?

d. Has *[child's name]* ever shared with you anything about the amount of time they have to eat? If so, can you talk about how they feel about the amount of time they have to eat?

3. (*If child doesn't eat the lunch every day*) how do you decide which days to eat the school lunch?

[ALL RESPONDENTS WHOSE CHILDREN EAT <5 DAYS of SCHOOL LUNCH]

4. Where does your child usually get lunch from on school days [on days they don't eat school meals]?
    a. *Probe about: lunch from home, store or restaurant, eat what a friend brings*

Now I'd like to hear a little bit about <u>your personal impressions</u> of the school lunch.

5. Other than from your child, how do you learn about the school lunch foods currently available at *[child's name]'s* school?
    a. Can you tell me about the communication you've gotten from your school about the school lunch this year, including how you received this information (such as emails, mailings, texts, or social media)?
        i. How do you know what is on the menu?
        ii. How would you describe your awareness about what is being served for lunch at [child's name]'s school and how the meals are served?
        iii. Is there other information you would like about the school meal program that you're not currently receiving?

6. What is your impression of school lunches at your child's school?
    a. *Probe about meal quality, healthfulness, quantity, and overall appeal*
    b. Are your impressions of the school lunch different now than they were before COVID?
    c. Any other impressions about school lunch you'd like to share?

Now, we will be talking about school lunch more generally.

d. What do you think are some of the benefits of schools providing lunch? What are some benefits of providing school lunch at no cost to all families?
    i. *Probe about family finances, schedules, food buying and food preparation work, child's health, social, or academic experiences at school*

e. *(if participating)* What do you think are the greatest benefits of school meals to your family? How about to others in your school community?
    i. Do you think your child would have anything more or different to say?

f. Do you think other families at your child's school feel differently from you about the school lunch?
    i. If so, in what way?

7. What do you think would be the most important way to encourage more children and families to participate in school lunch?
    a. *[If not already discussed]* Can you tell me whether you think there is any embarrassment or shame students or families feel about eating school lunch at *[child's name]*'s school? If this is something you or *[child's name]* have experienced, can you please tell me about it?
        i. Can you describe any ways this may have changed now that all students have been receiving free meals for the past two years?

Now I'd like to turn to challenges you and/or [*child's name*] may be experiencing with school lunch

8. Can you tell me about any difficulties you or your child may have experienced with the school lunch this current year or any things you or your child don't like about it that you haven't already told me?
    a. Can you tell me anything you've heard about your school experiencing supply chain or other COVID-related issues this year?
        i. [*If yes*] How would you say this has affected the school lunch?
9. Do you think your child's school provides meals that meet students' and families' differing needs? For example, are foods served at school similar to the kinds of foods you eat at home?
    a. If your student has any dietary restrictions for religious, health or other reasons, like Halal, vegan, nut or gluten-free, can you tell me how well you feel the school meets your child's needs?

10. Prior to COVID, how did your school ask families to provide information about whether you qualified for free or reduced-price school meals? Can you tell me what you know or remember about that process?

    a. [*If there was an application process*] Can you talk about how easy or difficult it was for you to complete the application?
    b. How did you feel about completing the application? Was it convenient/inconvenient, did you worry about sharing the information, did you feel bad or good about giving the school this information about your family, etc.
    c. Are there things your *[child's name]'s* school does that makes this work well?
    d. Are there things you wish your *[child's name]'s* school did to make this work better?
    e. What is your understanding about the benefits of filling out the application? (*Probe about perceived benefits to the family AND school*)

11. Before our meeting today, can you tell me what you had heard about the State of Maine continuing to provide school meals at no cost to all students next year, even after the federal program that has been doing this for the past two years expires?

a. Are you aware that the school meal applications are also used to determine many other types of funding for students like transportation, discounted internet, free Advance Placement testing, and P-EBT (pandemic electronic benefits transfer)
b. Did you know that submitting a school meal application also helps your school receive additional funding from the state and federal government?
c. Now that you know, what messaging do you think would helpful to explain the importance of submitting free and reduced price meal or other types income-related applications to other parents?
a. What factors do you think would motivate families to complete these application forms?
a. What do you think of the terms "Universal School Meals" or "Free Meals for All Students"? Can you tell me whether you have heard them before and what you think of them?

<u>School breakfast</u>

12. Does [child's name]'s school offer a school breakfast?
[If yes] Remind me how often *[child's name]* eats breakfast at school in the current year?
[If no, skip to Q13]
    a. Can you tell me why *[child's name]* eats breakfast or doesn't eat breakfast *[if they said never]?*
        i. Can you share any thoughts about the school breakfast –what you like about it–that are different from the things you told me about lunch?
        ii. Can you share any thoughts about the school breakfast –what you don't like about the school breakfast– that are different from things you don't like about the school lunch?
    b. When and where is breakfast served? (*Probe: before school, after the bell, in the classroom, school doesn't provide breakfast*)
    c. Where does your child usually get breakfast from on school days [on days they don't eat school breakfast]?
        i. Probe about: breakfast from home, store or restaurant
    d. Does your child get breakfast at school more or less often than prior to the pandemic?

[For children who eat school meals <5 days per week]

13. What is the main reason *[child's name]* doesn't eat the school meals more often?
    a. How do you think *[child's name]* would answer that question?

(All families)

14. What one thing could your school do to make you more excited about *[child's name]* eating school meals (breakfast and/or lunch)?
    a. How do you think *[child's name]* would answer that question?
